# Junctional adhesion molecule-like protein promotes tumor progression via the Wnt/β-catenin signaling pathway in lung adenocarcinoma

**DOI:** 10.1186/s12967-022-03457-w

**Published:** 2022-06-07

**Authors:** Qian Wu, Rui Li, Qing-Xiang Wang, Meng-Yu Zhang, Ting-Ting Liu, Yi-Qing Qu

**Affiliations:** 1 Department of Pulmonary and Critical Care Medicine, Shandong Key Laboratory of Infectious Respiratory Diseases, Qilu Hospital of Shandong University, Jinan, 250012 China; 2 Department of Pulmonary and Critical Care Medicine, The Second Hospital of Shandong University, Jinan, 250033 China

**Keywords:** Junctional adhesion molecule-like protein, Lung adenocarcinoma, Invasion, Migration, Tumor progression

## Abstract

**Background:**

Lung adenocarcinoma (LUAD) is a heavy social burden worldwide. Because the mechanisms involved in LUAD remain unclear, the prognosis of LUAD remains poor. Consequently, it is urgent to investigate the potential mechanisms of LUAD. Junctional adhesion molecule-like protein (JAML), is recognized as a tumorigenesis molecule in gastric cancer. However, the role of JAML in LUAD is still unclear. Here we aimed to evaluate the role of JAML in LUAD.

**Methods:**

qRT-PCR, Western blotting and immunohistochemistry were conducted to investigate the expression of JAML in LUAD tissues. JAML was knocked down and overexpressed in LUAD cells using transient transfection by siRNA and plasmids or stable transfection by lentivirus. Proliferation potential of LUAD cells were detected by Cell Counting Kit-8, EdU incorporation and Colony formation assay. Migration and invasion abilities of LUAD cells were determined by wound healing, transwell migration and invasion assays. Cell cycle and cell apoptosis were detected by flow cytometry. The effects of JAML in vivo were studied in xenograft tumor models. Western blotting was used to explore the molecular mechanisms of JAML function. In addition, rescue experiments were performed to verify the possible mechanisms.

**Results:**

JAML expression was elevated in LUAD tissues compared with peritumor tissues, and this upregulation was positively related to pT and pTNM. Furthermore, both in vitro and in vivo, JAML silencing markedly repressed malignant behaviors of LUAD cells and vice versa. Knockdown of JAML also mediated cell cycle arrest at G_0_/G_1_ phase and promoted apoptosis in LUAD cells. Mechanistically, silencing JAML repressed the process of epithelial-mesenchymal transition by inactivating the Wnt/β-catenin pathway in LUAD cells. Effects of JAML can be rescued by Wnt/β-catenin pathway activator in A549 cells.

**Conclusions:**

Our data reveal the oncogenic role of JAML in LUAD, indicating that JAML may be a predictive biomarker and novel therapeutic target for LUAD.

**Supplementary Information:**

The online version contains supplementary material available at 10.1186/s12967-022-03457-w.

## Introduction

Lung cancer is an extremely lethal disease worldwide [1]. Lung adenocarcinoma (LUAD), accounting for approximately 40% of all lung cancers, is the most common cancer [2]. Although important progress has been made in drug therapy, especially targeted drugs and immune check point inhibitors, the 5-year survival rate of LUAD is merely 15% [3]. The main reason for this is that the mechanisms involved in LUAD remain unclear, so to improve the survival rate, it is critical to investigate the potential mechanisms of LUAD.

Investigators have paid attention to junctional adhesion molecules (JAMs) in recent years. JAMs belong to the immunoglobulin (Ig) superfamily, consisting of junctional adhesion molecule-A (JAM-A), junctional adhesion molecule-B (JAM-B), junctional adhesion molecule-C (JAM-C), junctional adhesion molecule-4 (JAM-4) and junctional adhesion molecule-like (JAML); among them, JAM-A is the most studied molecule. Multiple studies had shown that overexpression (OE) of JAM-A induced tumorigenesis in cancers such as lung cancer, nasopharyngeal cancer and oral squamous cell carcinoma [4–6]. Moreover, a tumor-suppressive role of JAM-A was observed in pancreatic cancer and renal cell carcinoma [7, 8]. Interestingly, both tumorigenesis and tumor-suppressive roles of JAM-A were reported in breast cancer [9, 10]. Based on these studies, the role of JAM-A in carcinogenesis is complex and diverse in different cancers [11]. However, there have been few studies on JAML, especially on the relationship between JAML and LUAD.

As a newly reported JAM, JAML was first reported in 2003, and consists of one transmembrane fragment, two extracellular Ig-like domains, and a cytoplasmic tail. Some T cells, monocytes, neutrophils and other multiple cell types can express JAML. JAML can enhance the adhesion and migration ability of endothelial/epithelial cells, thereby influencing inflammatory reactions [12–15]. JAML also plays key roles in wound healing and atherosclerosis occurrence [16, 17]. In gastric cancer, overexpressed JAML facilitated the proliferation and migration of gastric cancer cells [18]. Gastric adenocarcinoma represents 95% of gastric cancer cases. Both LUAD and gastric adenocarcinoma originate from epithelial tissues, indicating that there may be common or similar mechanisms in the pathogenesis between the two diseases. Recently, the role of JAML was reported in LUAD, they found that JAML expression was decreased in LUAD and that JAML expression was correlated with immune infiltrates [19, 20], but most of their conclusions were based on online databases, and the role of JAML may be very complex in cancers. The actual role of JAML in LUAD is still unclear.

In our present study, the role of JAML in LUAD was elucidated. Initially, we investigated the expression of JAML in LUAD, followed by functional experiments both in vitro and in vivo. We also explored the underlying mechanisms by which JAML affects tumor progression in LUAD. Our results reveal the critical role of JAML in the progression of LUAD.

## Materials and methods

### Human LUAD samples and cell lines

All tumor and peritumor tissues were surgically resected from LUAD patients at the Second Hospital of Shandong University (Jinan, China). Each patient had an independent pathological review-confirmed diagnosis of LUAD. All studies were performed under supervision and approved by the Ethics Committee of the Second Hospital of Shandong University (KYLL-2021(LW)085), and adhered to the Declaration of Helsinki. Informed consent was obtained from human subjects. The privacy rights of human subjects must always be observed.

The LUAD cell lines, including A549, H1299, PC9 and H1975, were purchased from Procell Life Science & Technology Co. Ltd. (Wuhan, China). All LUAD cells were cultured in complete medium (RPMI 1640 for A549, H1299, H1975 or DMEM for PC9, plus 10% FBS). Cells were cultured in a 37 °C incubator with a humidified 5% CO2 atmosphere.

### RNA extraction and qRT-PCR

Snap-frozen tissues were homogenized and extracted using TRIzol^™^ Reagent (Ambion, 155596-026) following the manufacturer’s instructions. The concentration and purity of the RNA were determined by a DeNovix spectrophotometer. cDNA was synthesized by Hiscript ^®^ III RT SuperMix for qPCR (Vazyme, R323-01). Real-time PCR was performed on a Bio-Rad CFX Connect^™^ Real-Time System using a SYBR Green Premix Pro Taq HS qPCR Kit (ACCURATE BIOLOGY). Fold change was calculated as 2^–ΔΔCT^. PCR parameter settings were shown in Table 1. The melting curve data were collected to ensure PCR specificities. The primers used for PCR were shown in Table 2.

**Table 1:** PCR parameter settings

Temperature	Time	
95 °C	30 s	
95 °C	10 s	45 cycles
59 °C	30 s	
Melting Curve 65 °C, to 95 °C, increment 0.5 °C

**Table 2:** Primer sequences for PCR are as follows

Primer	Sequences
HOMO-JAML-F	5′-AGAGCACGCCAAGGACGAATA-3′
HOMO-JAML-R	5′-GGAGCAGGAGAGAGCCATCAT-3′
HOMO-GAPDH-138F	5′-GCACCGTCAAGGCTGAGAAC-3′
HOMO-GAPDH-138R	5′-TGGTGAAGACGCCAGTGGA-3′

*F* Forward, *R* Reverse

### Western blotting

Total proteins were extracted with RIPA buffer (Solarbio, #R0020) which added protease inhibitors (Solarbio, #P0100) and phosphatase inhibitors (Solarbio, #P1260). The lysates were separated on SDS-PAGE gels under constant voltage. After gel electrophoresis, the proteins were immediately trans-blotted to a Millipore PVDF membrane (0.45 µm) under constant current. 5% nonfat dry milk dissolved in TBST was used for blocking the blots for 1 h at room temperature (RT), and thus the blots were incubated with primary antibodies overnight at 4 °C. Next day, secondary antibodies were incubated with the blots for 1 h after extensive washing with TBST. All blots were detected by ECL reagent (Millipore) using a Tanon System for visualization after washing. β-actin expression was used to quantify the data. ImageJ software was used for the Western blotting analysis (version 5.2.1).

### Immunohistochemistry (IHC)

Slides (4 μm) were dewaxed in transparency agent and rehydrated in ethanol prior to antigen retrieval. Antigen retrieval was conducted by steaming slides in 1X EDTA buffer (Beyotime, #P0085) for 15 min by microwave irradiation (500 W). Endogenous peroxidase was quenched by treating samples with 3% H_2_O_2_ for 10 min. After the above steps, 5% goat serum (BOSTER, #16K03A09) was added onto slides for blocking, 30 min later, followed by staining in primary antibodies overnight at 4 °C. After a 1 h rewarming phase, secondary antibody was applied prior to DAB treatment (Zhongshanjinqiao, #ZLI-9108) for 1 h at RT. Hematoxylin was used for counterstain, then slides rinsed in running water for 5 min and differentiated with 1% hydrochloric acid alcohol for 2 s until the nuclei returned to blue. The tissues were dehydrated with graded ethanol and mounted with neutral resin (BOSTER, AR0038). Images were photographed by inverted microscopy and analyzed using IPP software.

### Immunofluorescence assay

Immunofluorescence assay was carried out with cells grown on coverslips (NEST, 801009). Adherent cells were fixed with 4% paraformaldehyde (Solarbio, #P1110) at RT for 30 min. The slides were incubated with 0.1% Triton X-100 (Solarbio, T8200) for 20 min for permeabilization immediately following 5% BSA (Solarbio, SW3015) for 30 min for blocking. The slides were incubated with primary antibody overnight at 4 °C after washing 3 times in PBS. A diluted fluorescent-labeled secondary antibody (BOSTER, BA1127) was incubated with slides for 2 h at 37 °C after 3 washes with PBS. DAPI (Solarbio, # C0065) was used for DNA staining. After extensive washing, the slides were mounted in fluorescence-quenching glycerol (Beyotime, P0126) and visualized by fluorescence microscopy (Nikon, Ti2).

### Antibodies

The antibodies and dilution ratios used for Western blotting, IHC and immunofluorescence assays are listed in Table 3.

**Table 3:** The antibodies and dilution ratios used for experiments

Antibody	Company	Batch number	Dilution ratios	Application
JAML	abcam	ab183714	1:1000	Western blotting
β-Actin	CST	3700	1:1000	Western blotting
MMP2	abcam	ab92536	1:1000	Western blotting
MMP9	abcam	ab76003	1:1000	Western blotting
β-Catenin	abcam	ab32572	1:5000	Western blotting
cyclin D1	abcam	ab226977	1:1000	Western blotting
Bcl2	abcam	ab32124	1:1000	Western blotting
Bax	abcam	ab32503	1:1000	Western blotting
Survivin	abcam	ab76424	1:5000	Western blotting
E-cadherin	Affinity	AF0131	1:1000	Western blotting
N-cadherin	abcam	ab76011	1:5000	Western blotting
Vimentin	abcam	ab92547	1:1000	Western blotting
Anti-mouse IgG, HRP-linked Antibody	CST	7076	1:3000	Western blotting
Anti-rabbit IgG, HRP-linked Antibody	CST	7074	1:3000	Western blotting
JAML	Proteintech	21302–1	1:200	IHC
Ki-67	CST	9449	1:400	IHC
JAML	Proteintech	21302–1	1:200	Immunofluorescence
DyLight 488 Conjugated AffiniPure Goat Anti-rabbit IgG	BOSTER	BA1127	1:1000	Immunofluorescence

*IHC* immunohistochemistiy, *CST* cell signaling technology

### SiRNA and plasmid transfection experiment

SiRNAs and plasmids were obtained from GenePharma (Shanghai, China) and used for gene silencing or overexpression. The siRNA sequences are shown in Table 4. SiRNAs or plasmids were transiently transfected into LUAD cells by jetPRIME transfection reagent (Polyplus, France). The transfection efficiency was validated after 48–72 h of transfection through Western blotting. After 48 h, the transfected cells were collected for in vitro functional experiments except for in the colony formation assay.

**Table 4:** SiRNAs sequences used in JAML knockdown experiment

Name	Sense or antisense	siRNA sequence
Negative control siRNA	sense sequence	5′-UUCUCCGAAGGUGUCACGUTT-3′
	antisense sequence	5′-ACGUGACACGUUCGGAGAATT-3′
siJAML1 siRNA	sense sequence	5′-GGAAUUGUCUGUGCCACAATT-3′
	antisense sequence	5′-UUGUGGCACAGACAAUUCCTT-3′
siJAML132 siRNA	sense sequence	5′-GAGCACAGAAGACAAAUGUTT-3′
	antisense sequence	5′-ACAUUUGUCUUCUGUGCUCTT-3′
siJAML272 siRNA	sense sequence	5′-GGGACAUCUUAUGCAAUGATT-3′
	antisense sequence	5′-UCAUUGCAUAAGAUGUCCCTT-3′

### Lentivirus transfection experiment

The lentiviruses were obtained from GeneChem (Shanghai, China). Lentivirus sequences used for JAML knockdown are listed in Table 5. After transfected with lentivirus (multiplicity of infection, MOI: 10 for A549 and 20 for PC9) and selected with puromycin (2 mg/ml) for 7 days, stable cell lines were harvested. HitransG (Genechem, Shanghai, China) was used for better lentivirus infection. The transfection efficiency was analyzed by Western blotting. The stable cell lines were collected for colony formation assays and xenograft tumor models.

**Table 5:** The lentivirus sequences used in JAML knockdown experiment

NO	Accession	Target seq	CDS	GC%
JAML-	NM_153206	gaAGACTAATCCAGAGATAAA	30.1184	31.58%
RNAi_				
(102663)				
JAML-	NM_153206	ccCTGTTCTGATATTGATCGT	30.1184	36.84%
RNAi_				
(102664)				
JAML-	NM_153206	gtATTTCGTTACTACCACAAA	30.1184	31.58%
RNAi_				
(102665)				

Description: Homo sapiens junction adhesion molecule like (JAML), transcript variant 2, mRNA

*RNAi* RNA interference, *CDS* Coding sequence

### CCK8 and EdU assays

For the CCK8 assay, 100 μl of cell suspension containing 1500 cells was inoculated in 96-well plates, followed by culturing in a cell incubator. Twenty-four hours after seeding, the cell viability was measured using CCK8 reagent (MedChemExpress, #HY-K0301) following the manufacturer’s protocol. This was considered as Day 0. The proliferation rates at Day 0, 1, 2, 3 and 4 after seeding were detected. At each time point, 100 μl CCK-8 containing medium without FBS was replaced into every well for measurement 1 h later. OD450 was measured on a microplate reader (Bio–Rad Laboratories).

For the EdU assay, cells were precultured in medium containing 10 μmol/L EdU (RiboBio, #C10310) in 96-well plates for 2 h. Then, the EdU-containing medium was removed. Cells were fixed with 4% paraformaldehyde at RT for 30 min, and washed twice with PBS. After 0.5% Triton-100 for 20 min for permeabilization, Apollo and Hoechst dyes were used according to the manufacturer’s protocol. The number of Hoechst-stained cells (blue-fluorescent, total cells) and EdU-positive cells (red-fluorescent) in each field was recorded under a Nikon Eclipse Ti2 microscope. The percentage of EdU-positive cells was calculated using the formula below: Relative EdU rate % = (EdU-positive cells/total cells) × 100%.

### Colony formation efficiency assay

A total of 5 × 10^2^ cells were independently inoculated into six-well plates after resuspension. The plates were incubated in a 37 °C incubator for 2 weeks. Every 3 days, the complete medium was replaced in per well. On Day 15, visible colonies (diameter > 0.5 mm) were stained with 0.1% crystal violet (Solarbio, #G106) overnight after fixing with 4% paraformaldehyde for 20 min. After washing 3 times and air drying, monoclonal colonies were photographed by camera.

### Wound healing assay

LUAD cells used for wound healing assay were cultured in six-well plates and grown to 100% confluency. Artificial homogeneous wounds were made with a 200 μl pipette tip. After washing twice with PBS to remove debris, FBS-free medium was added to six-well plates. At 0 h and after incubation for an appropriate time in the cell incubator, the wounded areas were photographed under microscopy. The data were quantified using the following formula: Migration Rate % = (initial scratch area − scratch area at time t)/initial scratch area × 100%.

### Cell Transwell assays

8.0-µm transwell chambers (Corning, USA) in 24-well plates were used for transwell assays. A total of 5 × 10^4^ cells suspended in 200 μl FBS-free medium were seeded evenly onto Matrigel (Corning, 356234)-coated or Matrigel-uncoated top chambers for the invasion or migration assay, and 700 μl medium (1640 or DMEM plus 20% FBS) was added to the lower chambers. The plates were then returned to a 37 °C incubator for 24 h. Cotton swabs were used to wiped off non-migrated and non-invaded cells on the top layer, the migrated and invaded cells on the bottom of the chambers were fixed with 4% paraformaldehyde, subsequently stained with 0.1% crystal violet. Three random fields of stained cells were photographed by microscope (Nexcope).

### Cell cycle assay

Cells were harvested with 0.05% trypsin at 70–80% cell density and then washed twice with precooled PBS. The cell pellet was fixed with 70% cold ethanol at − 20 °C overnight. Within 3 days, the fixed cells were washed twice with precooled PBS, and resuspended in PBS containing propidium iodide (PI) (40 μg/ml, BB-4104) plus RNAase A (250 μg/ml, BB-4104). The mixtures were under a 37 °C water bath protected from light for 30 min before analysis. The distribution of cell cycle was detected by flow cytometry (BD FACSCalibur). Mod-Fit software (Verity Software House) was used to analyze different cell cycle phases.

### Cell apoptosis assay

Cells were grown to 70–80% confluency before experiments, and collected with EDTA-free trypsin. After washing twice with precooled PBS, the cell pellet was resuspended in 1X binding buffer, and then collected 1 × 10^5^ cells, 5 μl PI and 5 μl Annexin V-FITC (Meilunbio, MA0220-2) were added into binding buffer. The samples protected from light for 15 min at RT. Flow cytometry was carried out to record the apoptosis rate, thus the data were analyzed using FlowJo software.

### Xenograft transplantation in vivo

The animal study was approved by Ethics Committee of the Second Hospital of Shandong University (KYLL-2021(LW)086). All animal experiments complied with the ARRIVE guidelines. All experiments were handled according to U.K. legislation (1986) Animals (Scientific Procedures) Act. 4 weeks old female athymic nude mice (BALB/c mice) were acquired from Vital River Company (Beijing, China). Animals were randomly assigned to each group before start. Lentivirus-transfected LUAD cell lines were resuspended on ice with precooled PBS. A total of 1 × 10^7^ cells were subcutaneously injected into right armpit areas of mice for tumorigenesis. At the end point, the tumors were excised, followed by preparation for IHC staining. The tumor volume was calculated with the following formula: volume (mm^3^) = 1/2 (length (mm) × width^2^ (mm^2^)).

### Statistical analysis

GraphPad Prism version 6 (GraphPad Software, USA) was used for data statistical analysis and drawing graphics. The data are expressed as the mean ± SEM for all groups. A *P* value of < 0.05 was considered statistically significant.

## Results

### JAML expression is elevated in human LUAD tissues

**Fig. 1:**
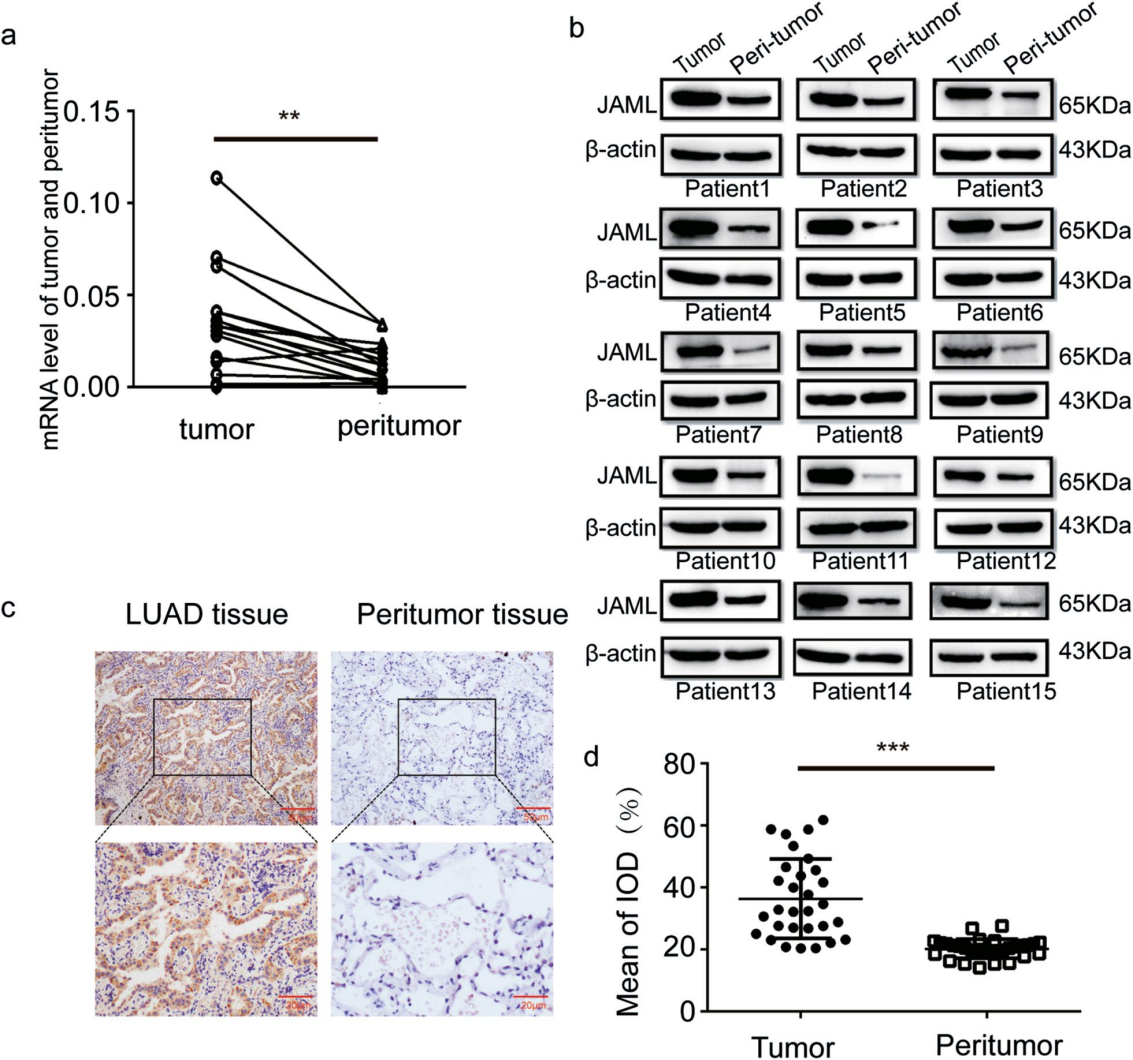
JAML expression is elevated in human LUAD tissues. **a**, **b** The expression of JAML was elevated in 15 pairs of LUAD tissues detected by qRT-PCR (**a**) and Western blotting (**b)**. **c** Representative IHC images of JAML expression in 30 pairs of LUAD tissues. **d** Mean of IOD for JAML in 30 pairs of LUAD tissues. *P < 0.05, **P < 0.01, ***P < 0.001

The expression level of JAML was analyzed by qRT-PCR and Western blotting in 15 pairs of LUAD tissues. Our results demonstrated that JAML expression was significantly elevated in tumor tissues when compared with peritumor tissues (Fig. 1a, b). IHC analysis confirmed the results that expression of JAML in tumor tissues was significantly upregulated in 30 pairs of LUAD tissues (Fig. 1c, d). The clinicopathological parameters of the patients were listed in Table 6. The samples were sorted by the median value of JAML according to the IHC scores into high and low JAML expression groups. High JAML expression was significantly correlated with advanced pT stage and pTNM stage, but not to age, sex, the pN stage, or the pM stage (Table 6). In summary, the results above imply that expression of JAML is elevated in LUAD, and JAML may plays a role as an oncogene in LUAD, Table 6.

**Table 6:** Correlation between JAML expression and clinicopathological features in LUAD

Factors	Sample	JAML expression		*P* value
		Low	High	
Age				
< 60	12	8	4	NS
≥ 60	18	7	11	
Gender				
Male	11	7	4	NS
Female	19	8	11	
pT status				
T1	16	13	3	*P* < 0.001***
T2-4	14	2	12	
pN status				
N0	24	14	10	NS
N1 + 2	6	1	5	
pM status				
M0	27	14	13	NS
M1	3	1	2	
pTNM stage				
I	14	12	2	*P* < 0.001***
II + III + IV	16	3	13	

The Chi-squared test or Fisher’s exact test was used for statistical analysis

*T* tumor, *N* node, *M* metastasis, *NS* no significance

***P < 0.001

### JAML overexpression promotes LUAD cell proliferation

**Fig. 2:**
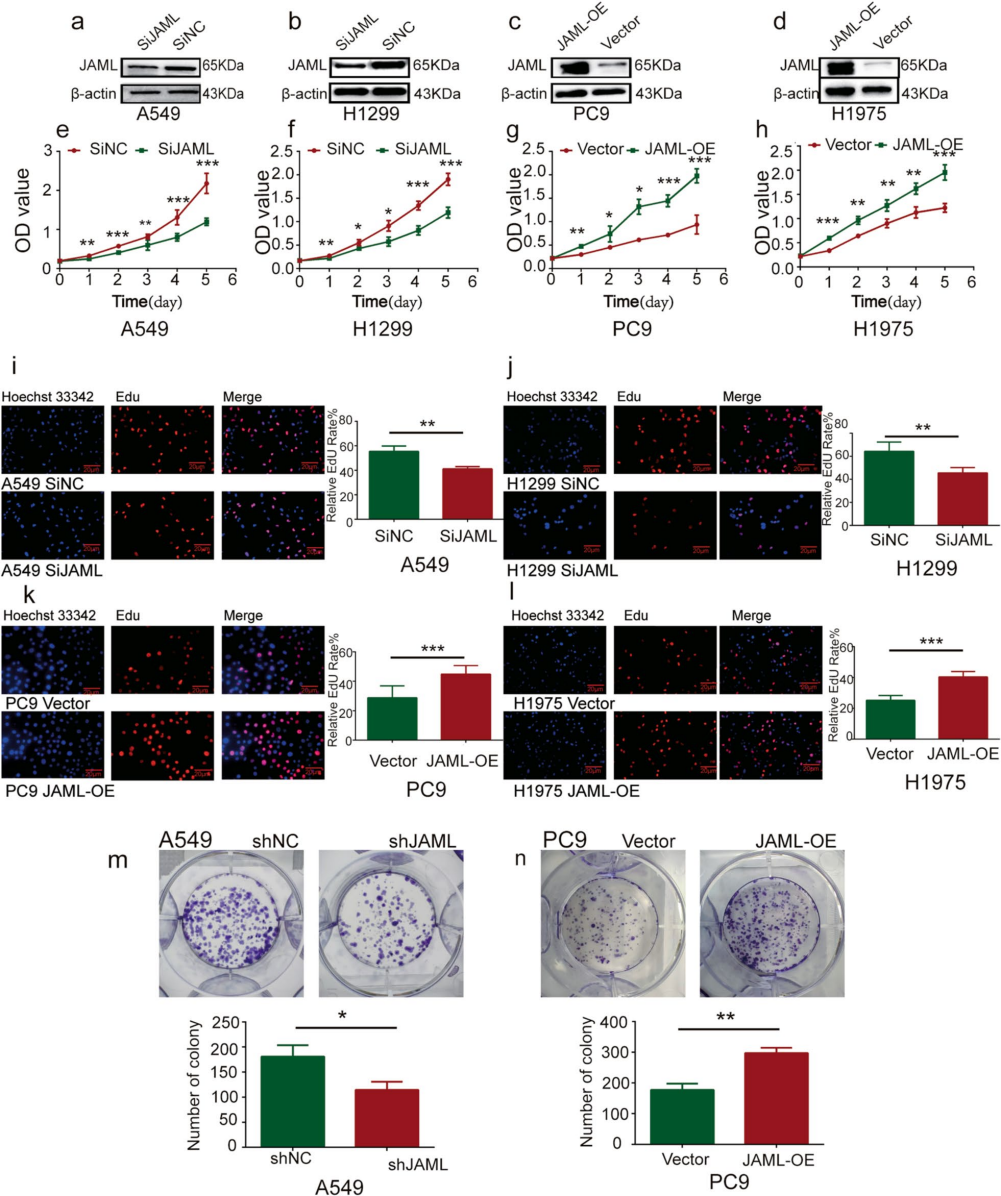
JAML overexpression promotes LUAD cell proliferation. **a**–**d** The efficiency of JAML knockdown in A549 (**a**) and H1299 cells (**b**) or overexpression in PC9 (**c**) and H1975 cells (**d**) were confirmed by Western blotting. The internal reference gene was β-actin. **e**–**h** Cells growth of A549 (**e**), H1299 (**f**), PC9 (**g**) and H1975 cells (**h**) with JAML knockdown or overexpression was analyzed with CCK8 assay. *P < 0.5, **P < 0.01, ***P < 0.001, compared with SiNC or Vector group. **i**–**l** Cell proliferation in A549 (**i**) and H1299 cells (**j**) after JAML knockdown and in PC9 (**k**) and H1975 cells (**l**) after JAML overexpression was observed through EdU assay, quantitative analysis n = 3, unpaired *t* test, **P < 0.01, ***P < 0.001, compared with SiNC or Vector group. **m**, **n** The colony formation showed that cell growth potential was inhibited in A549 cells (**m**) following JAML knockdown, and that cell growth potential was increased in PC9 cells (**n**) following JAML overexpression (top panels). The number of foci was shown in the bottom panels. Quantitative analysis n = 3, unpaired *t* test, *P < 0.05, compared with the ShNC group, **P < 0.01, compared with the vector group

The endogenous expression of JAML in four LUAD cell lines (A549, H1299, PC9 and H1975) was detected by Western blotting, the result revealed that JAML was relatively upregulated in A549 and H1299 cells, and that JAML was relatively downregulated in PC9 and H1975 cells (Additional file 1: Fig. S1a, S1b). A549 and H1299 cells were chosen for JAML knockdown study. PC9 and H1975 cells were chosen for JAML overexpression study. Immunofluorescence assay showed that JAML was expressed in cytoplasm of LUAD cells (Additional file 1: Fig. S1c). SiJAML1 sequence was the most effective siRNA in A549 and H1299 cells analyzed by Western blotting, and was used in subsequent functional experiments. The overexpression efficiency of plasmids was also evaluated via Western blotting (Additional file 1: Fig. S1d-g, Fig. 2a–d). CCK8, EdU and colony formation assays were conducted to determine the effect of JAML on proliferation in LUAD cells. CCK-8 and EdU assays showed that JAML knockdown inhibited proliferation in A549 and H1299 cells and that JAML overexpression promoted proliferation in PC9 and H1975 cells (Fig. 2e–l). A clone formation assay also verified that JAML knockdown repressed the proliferation of A549 cells and that JAML overexpression facilitated the proliferation of PC9 cells (Fig. 2m, n). The results above indicate that JAML overexpression can promote the proliferation of LUAD cells.

### JAML overexpression enhances migration and invasion in LUAD cells

**Fig. 3:**
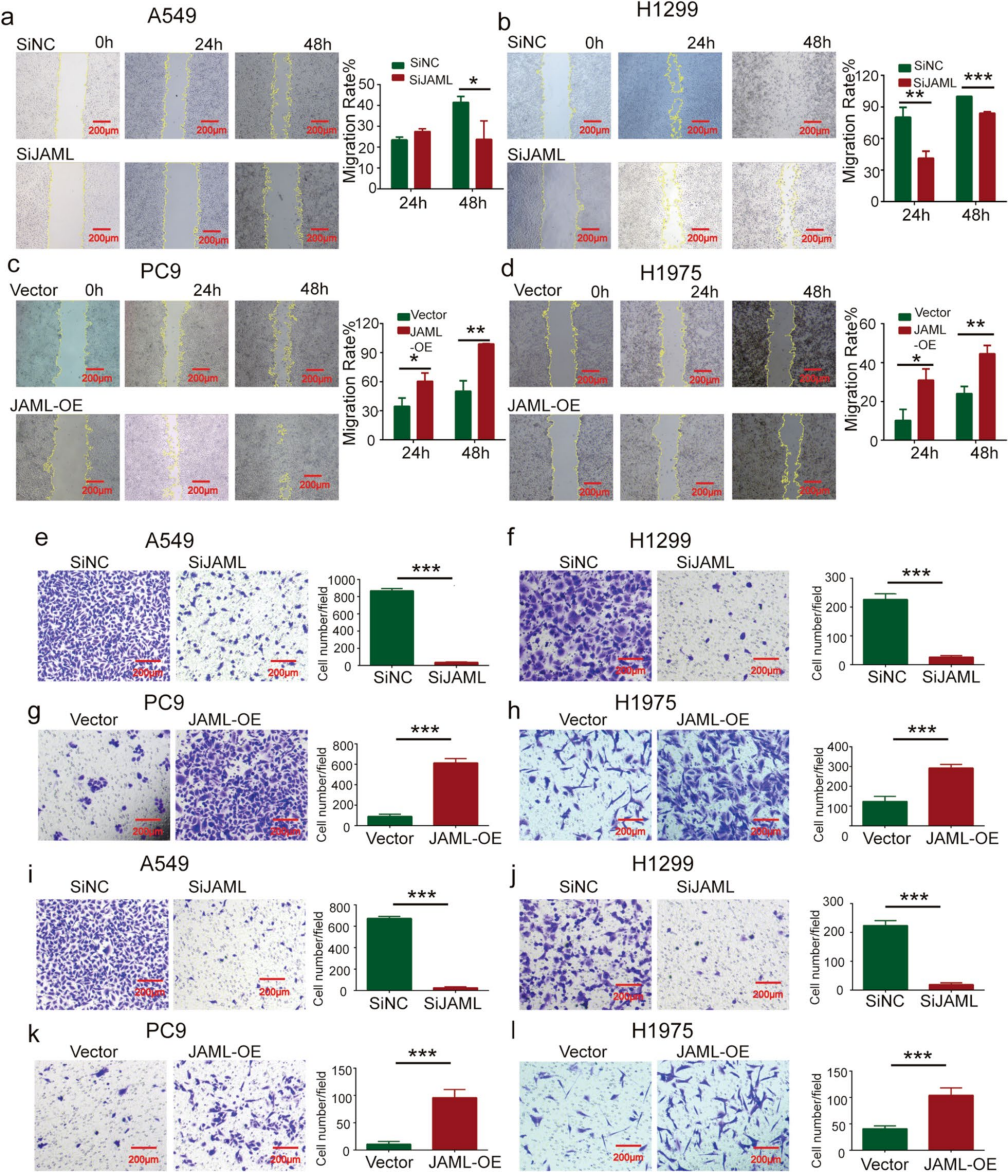
JAML overexpression promotes migration and invasion in LUAD cells. **a**–**d** The wound healing assay indicated that JAML knockdown suppressed the migratory capabilities of A549 (**a**) and H1299 cells (**b**), and that JAML overexpression promoted the migratory capabilities of PC9 (**c**) and H1975 cells (**d**). Representative images of wound healing assays were obtained (left panel). ImageJ software was used to measure the migration rate (right panel). n = 3, unpaired* t* test, *P < 0.05, **P < 0.01, ***P < 0.001, compared with the SiNC or vector groups. **e**–**h** The transwell migration assay confirmed that JAML knockdown suppressed the migratory capabilities of A549 **e** and H1299 cells (**f**), and that JAML overexpression promoted the migratory capabilities of PC9 (**g**) and H1975 cells **h**. Representative images of transwell migration assays were obtained (left panel). ImageJ software was used to count the number of migrated cells (right panel). n = 3, unpaired* t* test, ***P < 0.01, compared with the SiNC or vector groups. **i**–**l** Transwell invasion assays indicated that the invasion ability was downregulated in A549 (**i**) and H1299 (**j**) cells when JAML was knocked down, and that the invasion ability was upregulated in PC9 (**k**) and H1975 cells (**l**). Representative images of transwell invasion were obtained (left panel). ImageJ software was used to count the number of invaded cells from 3 randomly selected fields (right panel). n = 3, unpaired* t* test, ***P < 0.001, compared with the SiNC or vector groups

Wound healing assays and transwell migration and invasion assays were performed to investigate the effect of JAML on migration and invasion in LUAD cells. Silencing JAML in A549 and H1299 cells inhibited migration in the wound healing assays. Conversely, overexpression of JAML in PC9 and H1975 cells promoted migration in the wound healing assays (Fig. 3a–d). JAML knockdown in A549 and H1299 cells can inhibited migration and invasion in the transwell assays, in contrast, the opposite results were observed in PC9 and H1975 cells with overexpressed JAML (Fig. 3e–l). These results above indicate that JAML overexpression could facilitate migration and invasion capacities in LUAD cells.

### JAML knockdown induces cell cycle arrest and promotes cell apoptosis in LUAD cells

**Fig. 4:**
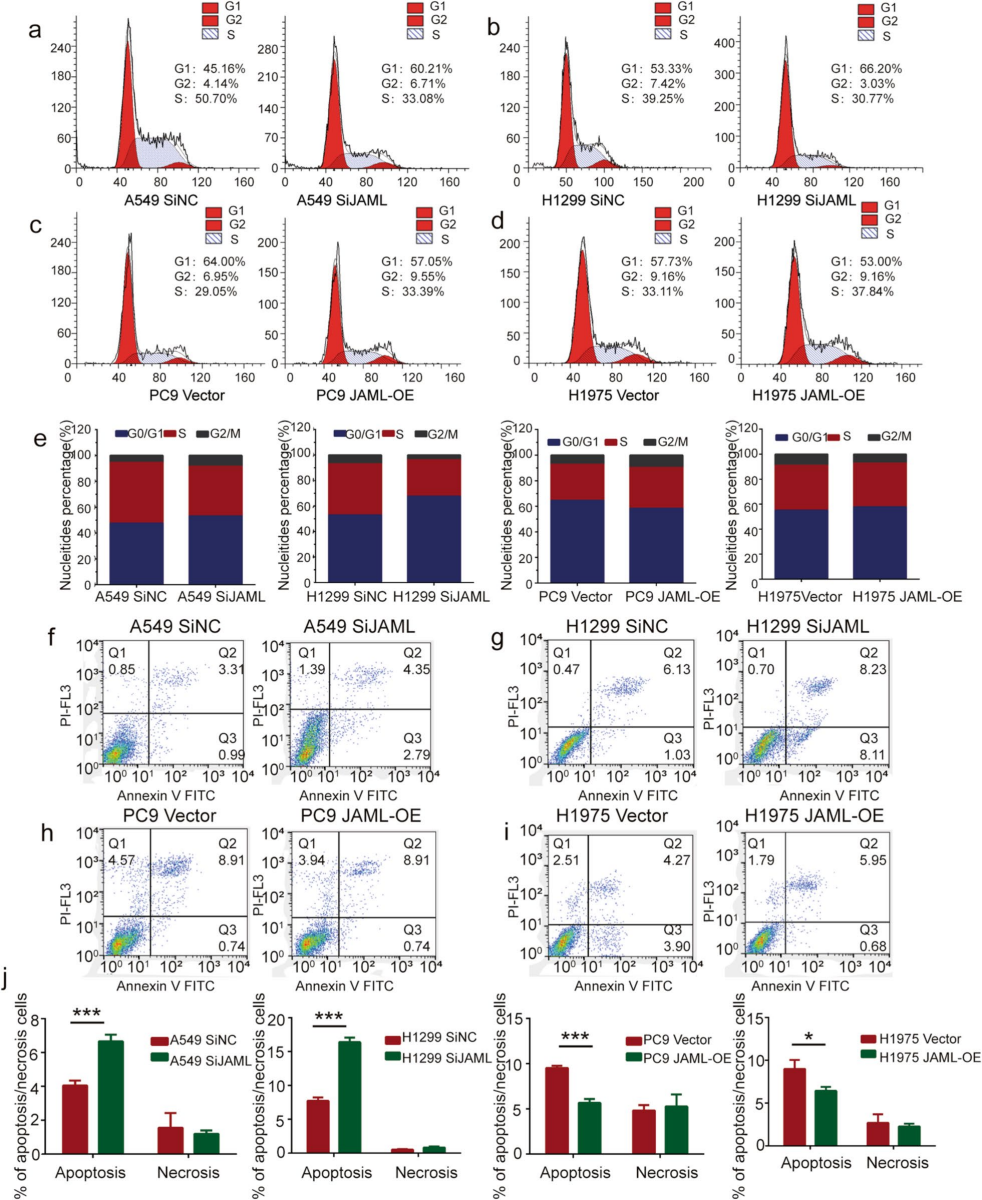
JAML knockdown mediates cell cycle arrest and promotes cell apoptosis in LUAD cells. **a**–**e** The percentages of cells in the different cycle phases were showed after JAML knockdown in A549 (**a**) and H1299 cells (**b**) and JAML overexpression in PC9 (**c**) and H1975 cells (**d**). Superimposed histograms of different cycle phases are shown in (**e**). **f**–**j** Cell apoptosis was analyzed by staining with Annexin V-FITC and PI after JAML knockdown in A549 (**f**) and H1299 cells (**g**) and JAML overexpression in PC9 (**h**) and H1975 cells (**i**). Histograms of the results from different apoptotic cells is displayed on (**j)**

Flow cytometry was performed to evaluate the effects of JAML on the cell cycle and cell apoptosis in LUAD cells. Our results showed that silencing JAML increased G_0_/G_1_ phase cells and decreased S + G_2_/M phase cells in A549 and H1299 cells. Overexpression of JAML was significantly associated with a greater proportion at the S + G_2_/M phase at the expense of the G_0_/G_1_ phase in PC9 and H1975 cells (Fig. 4a–e, Table 7). Cell apoptotic rate was elevated when JAML was knocked down in A549 and H1299 cell. Conversely, the cell apoptotic rate was decreased when JAML was overexpressed in PC9 and H1975 cells(Fig. 4f–j, Table 8). These results reveal that JAML knockdown can induce cell cycle arrest in G_0_/G_1_ phases and promote cell apoptosis in LUAD cells.

**Table 7:** FACS analysis of the distribution of cells in the different cycle phase

Group	Percentage of cells	
	G_0_/G_1_	S + G_2_/M
A549 SiNC^1^	46.08 ± 0.77	53.92 ± 0.77
A549 SiJAML^1^	56.68 ± 2.03^**^	43.32 ± 2.03^**^
H1299 SiNC^1^	52.65 ± 0.53	47.35 ± 0.53
H1299 SiJAML^1^	65.77 ± 1.23^**^	34.23 ± 1.23^***^
PC9 Vector^2^	64.65 ± 0.50	35.36 ± 0.50
PC9 JAML-OE^2^	58.36 ± 0.67^***^	41.64 ± 0.67^***^
H1975 Vector^1^	57.38 ± 0.17	42.62 ± 0.17
H1975 JAML-OE^1^	54.54 ± 0.84^*^	45.46 ± 0.84^*^

Quantitative analysis^1^: n = 3, ^2^: n = 4, unpaired *t* test

*P < 0.05

**P < 0.01

***P < 0.001 compared with SiNC or vector group

**Table 8:** FACS analysis of cell apoptotic rates

	Apoptosis	Necrosis
A549 SiNC	4.06 ± 0.17	1.56 ± 0.50
A549 SiJAML	6.67 ± 0.22***	1.20 ± 0.11
H1299 SiNC	6.75 ± 0.32	0.53 ± 0.052
H1299 SiJAML	16.39 ± 0.39***	0.84 ± 0.08
PC9 vector	9.53 ± 0.14	4.83 ± 0.35
PC9 JAML-OE	5.66 ± 0.25***	5.27 ± 0.77
H1975 vector	9.00 ± 0.60	2.70 ± 0.57
H1975 JAML-OE	6.45 ± 0.25*	2.28 ± 0.19

Quantitative analysis n = 3, unpaired *t* test

*P < 0.05

***P < 0.001 compared with SiNC or vector group

### JAML acts as an oncogene in LUAD cells in vivo

**Fig. 5:**
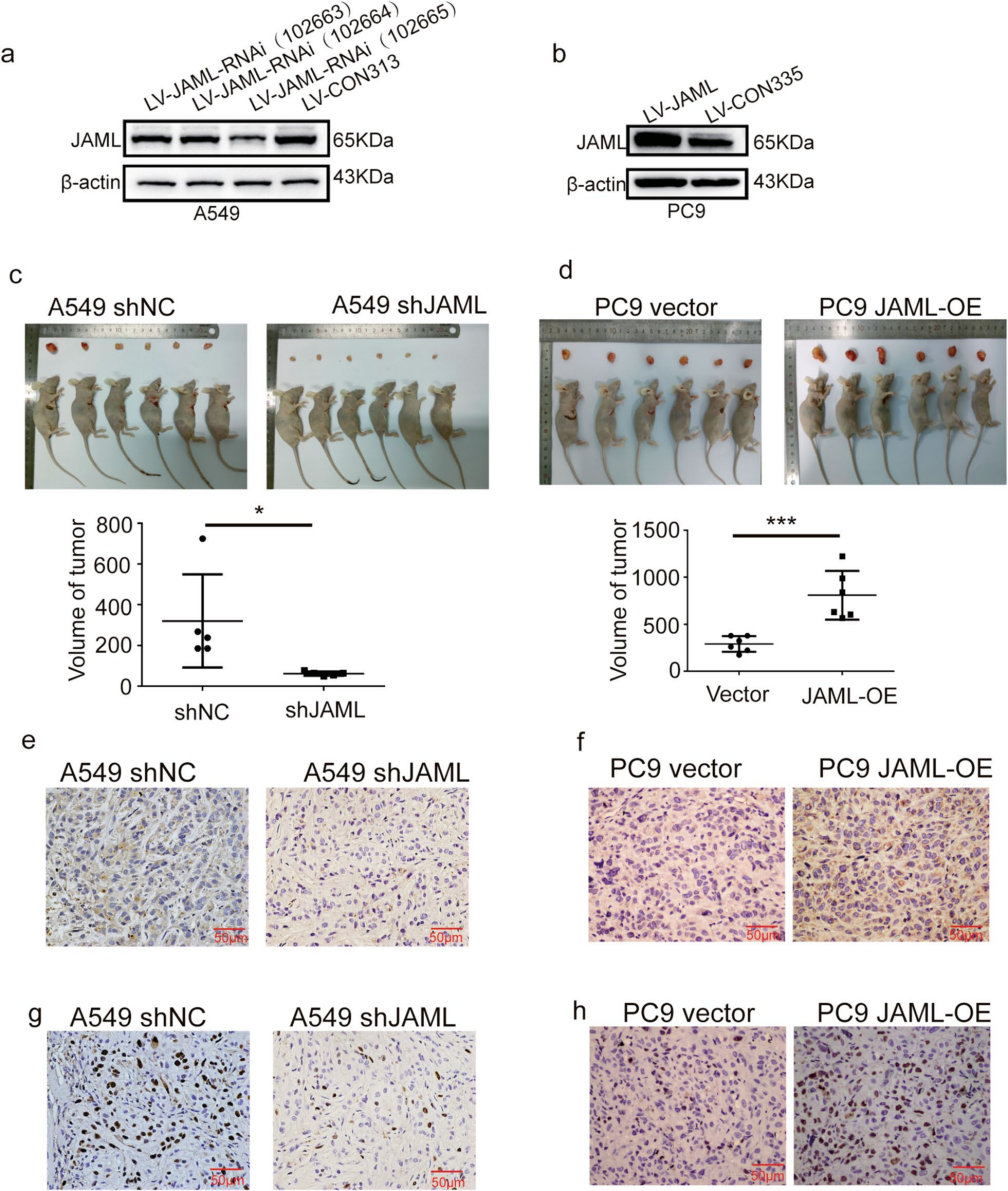
JAML overexpression promotes LUAD cell proliferation in vivo. **a**, **b** Knockdown or overexpression efficiency of JAML was confirmed in A549 (**a**) or PC9 cells (**b**) after stable transfection of lentivirus by Western blotting. The internal reference gene was β-actin. **c**, **d** Representative images of tumors formed by A549 (**c**) or PC9 cells (**d**) 5 weeks after subcutaneous injection, quantitative analysis n = 6, unpaired *t* test, *P < 0.05, ***P < 0.001, compared with the ShNC or vector groups. **e**, **f** Representative images of IHC staining for JAML in xenograft tumors caused by A549 (**e**) or PC9 cells (**f**). **g**, **h** Representative images of IHC staining for Ki67 in xenograft tumors caused by A549 (**g**) and PC9 cells (**h) **

JAML expression modulated tumor formation in xenograft transplantation experiments. A549 cells transfected with either a control shRNA or shRNAs specifcally against JAML were detected by Western blotting, and the JAML-RNAi (102665) was the most efective inhibitor of JAML and was chosen for next experiments (Fig. 5a). Stably transfected PC9 cell lines was corfirmed by Western blotting (Fig. 5b). Four groups of nude mice were injected with A549-shNC, A549-shJAML, PC9-Vector, and PC9-JAML-OE cells. As expected, compared with the shNC or vector group, JAML knockdown significantly inhibited the xenograft tumor growth of A549 cells (Fig. 5c), and overexpression of JAML promoted the xenograft tumor growth of PC9 cells (Fig. 5d). Alteration of JAML expression in the xenograft tumors were confirmed by IHC (Fig. 5e, f). JAML knock down inhibited Ki67 expression while JAML overexpress increased Ki67 expression compared with the shNC or vector group by IHC (Fig. 5g, h). Thus, the in vitro and in vivo experimental data collectively reveal the oncogenic property of JAML in LUAD cells.

### Effect of JAML on MMPs, Bax/Bcl2 and EMT in LUAD cells

**Fig. 6:**
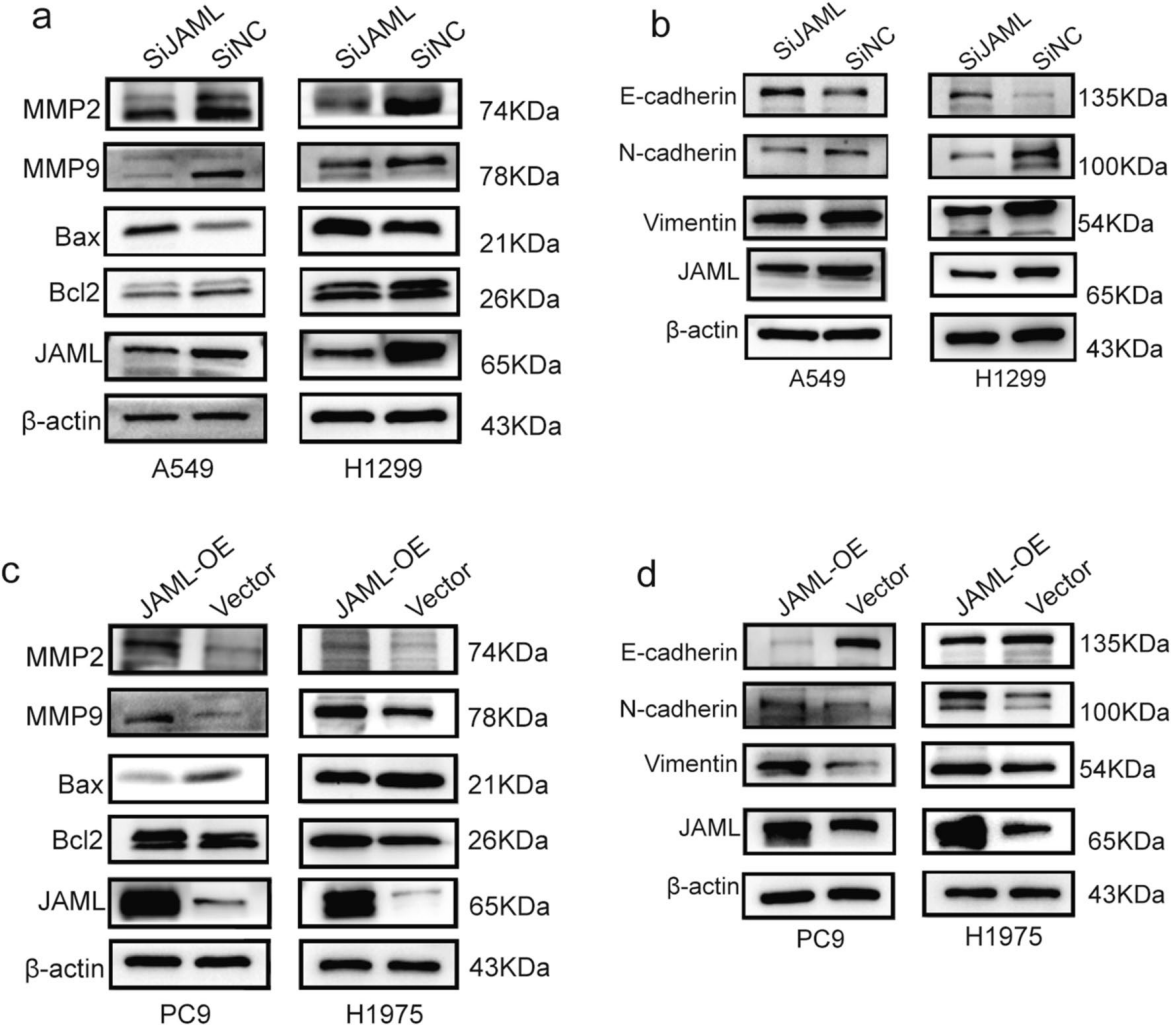
Effects of JAML on MMPs, Bax/Bcl2, and EMT in LUAD cells. **a**, **b** Expression of MMP2, MMP9, Bax, Bcl2 and EMT markers when JAML was knockdown in LUAD cells (A549, H1299). **c**, **d** Expression of MMP2, MMP9, Bax, Bcl2 and EMT markers when JAML was overexpressed in LUAD cells (PC9, H1975). The internal reference gene was β-actin

Based on the results of the above functional phenotypic studies, molecular mechanisms of JAML functions were further detected by Western blotting. Matrix metalloproteinase 2 (MMP2) and Matrix metalloproteinase 9 (MMP9) are important for tumor cell proliferation. Our results showed that MMP2 and MMP9 were downregulated when JAML was knocked down in A549 and H1299 cells (Fig. 6a). Bax/Bcl2 are key proteins related to apoptosis. Knockdown of JAML in A549 and H1299 cells significantly decreased Bcl2 with a concomitant upregulation of Bax (Fig. 6a). Additionally, the epithelial-to-mesenchymal transition (EMT) is a common step in tumor progression for tumors of epithelial origin. Our results showed that JAML knockdown led to N-cadherin and vimentin repression and E-cadherin induction in A549 and H1299 cells (Fig. 6b). Conversely, opposite results were obtained when JAML was overexpressed in PC9 and H1975 cells (Fig. 6c, d). These results demonstrated that the mechanisms of JAML-induced proliferation and JAML‐inhibited apoptosis and that JAML induced tumor progression through EMT in LUAD cells.

### JAML facilitates EMT partially by activating the Wnt/β-catenin signaling pathway in LUAD cells

**Fig.7:**
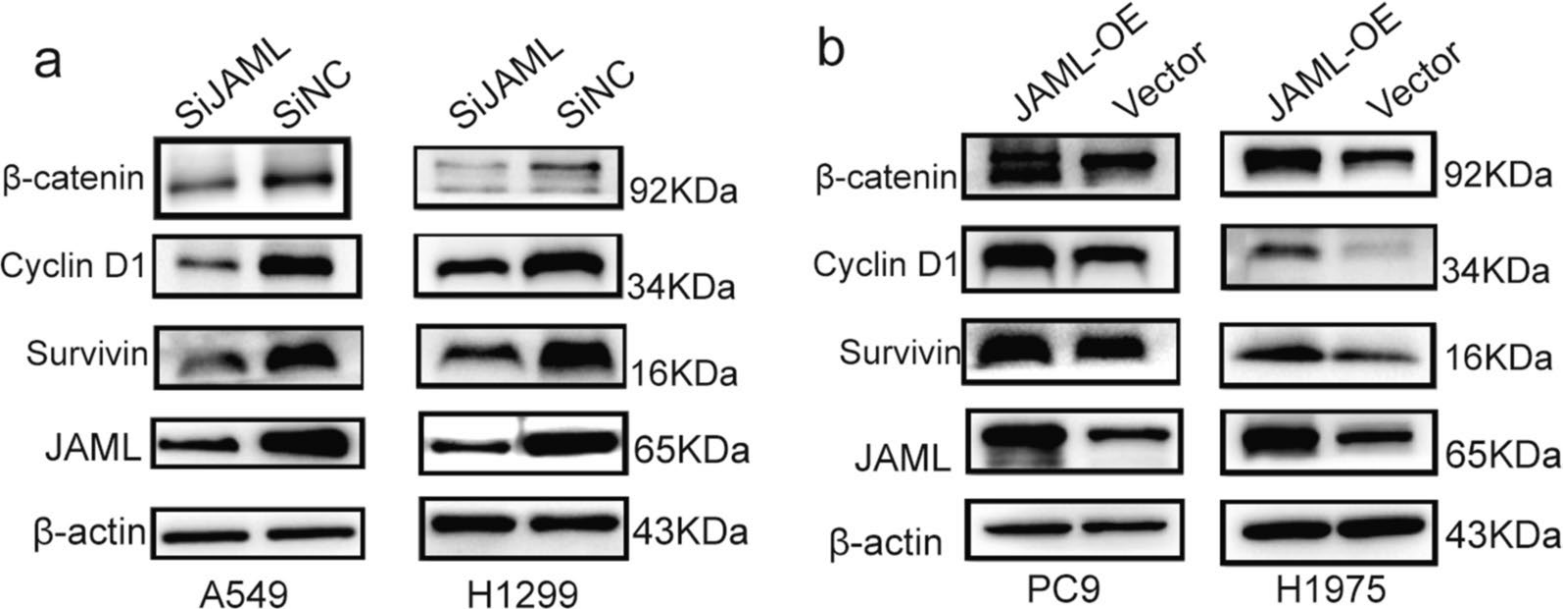
JAML facilitates EMT partially by activating the Wnt/β-catenin signaling pathway in LUAD cells. **a** Changes of key molecules in Wnt/β-catenin pathway when JAML was knockdown in LUAD cells (A549, H1299). **b** Changes of key molecules in Wnt/β-catenin pathway when JAML was overexpressed in LUAD cells (PC9, H1975). The internal reference gene was β-actin

Given the prominent role that β-catenin has been shown in EMT, interactions between JAML and β-catenin was further evaluated in LUAD cells. β-catenin was repressed in A549 and H1299 cells when silencing JAML (Fig. 7a). Conversely, β-catenin was elevated in PC9 and H1975 cells when JAML was overexpressed (Fig. 7b). These results indicate that JAML affects β-catenin expression, but the in-depth mechanism requires further exploration. Notably, cyclin D1 and survivin are the main effective molecules downstream of the Wnt/β-catenin pathway. Upon JAML knockdown, cyclin D1 and survivin were sharply reduced in A549 and H1299 cells (Fig. 7a), which might be responsible for cell cycle arrest and promote apoptosis. Conversely, cyclin D1 and survivin were elevated in PC9 and H1975 cells when JAML was overexpressed (Fig. 7b). Collectively, we supposed that JAML may facilitate EMT process in part through activating Wnt/β-catenin signaling pathway in LUAD cells.

### Effects of JAML can be rescued by Wnt/β-catenin activator in A549 cells

**Fig. 8:**
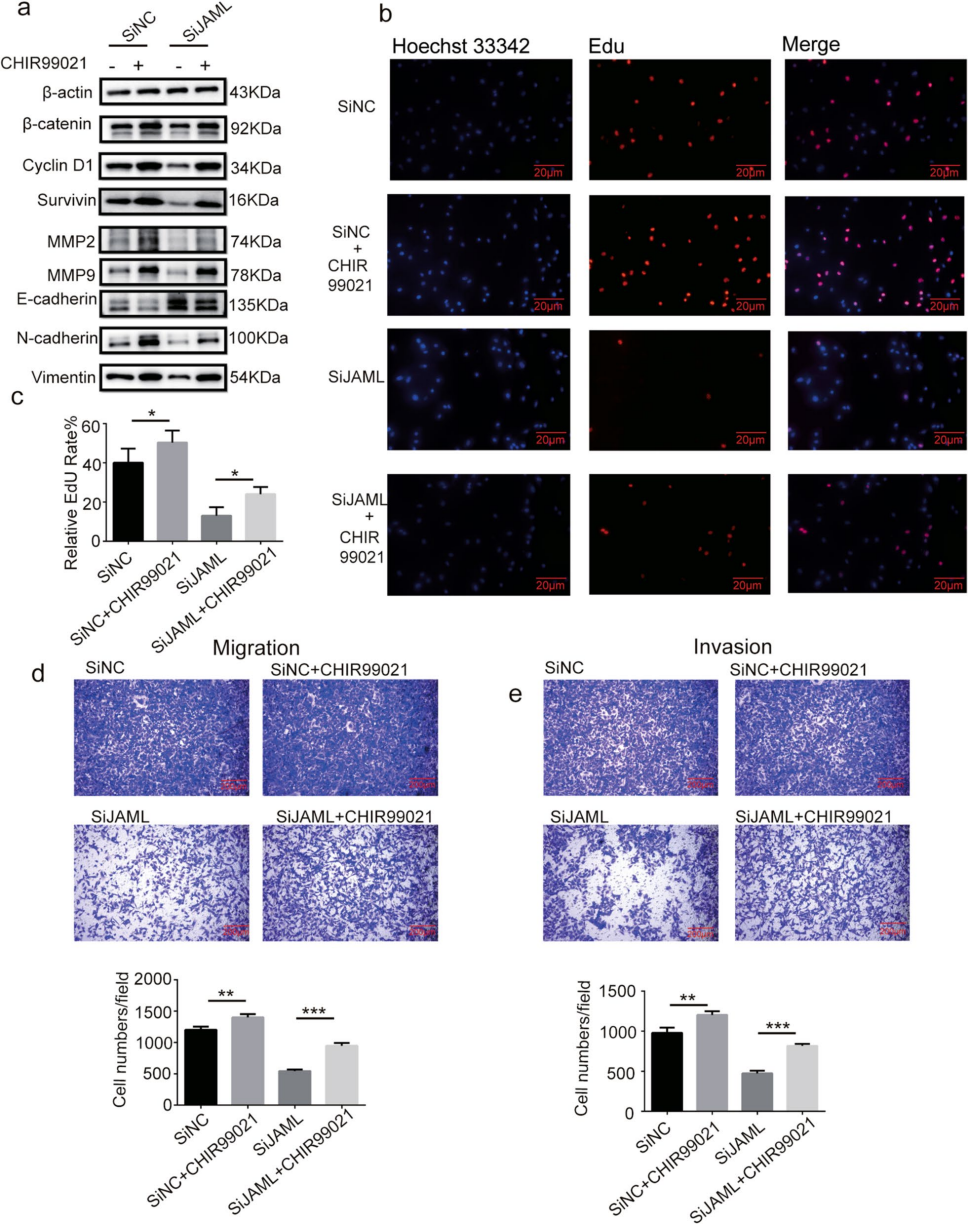
Effects of JAML can be rescued by Wnt/β-catenin activator in A549 cells. A549 cells with JAML knockdown was treated with Wnt/β-catenin activator (CHIR99021, 5 μM/ml) for 24 h. **a** The protein levels of MMP2, MMP9, EMT markers and key molecules of Wnt/β-catenin pathway (β-catenin, cyclin D1, survivin) were detected by Western blotting. The internal reference gene was β-actin. **b**, **c** EdU assay showed that repression of cell proliferation caused by JAML knockdown in A549 cells can be rescued by CHIR99021, quantitative analysis n = 3, unpaired *t* test, *P < 0.05, compared with CHIR99021(-) group. **d**, **e** Transwell assays showed that inhibition of migration (**d**) and invasion (**e**) abilities caused by JAML knockdown in A549 cells can be rescued by CHIR99021. Representative images of transwell were obtained (top panel). ImageJ software was used to count the number of migrated and invaded cells from 3 randomly selected fields (bottom panel), n = 3, unpaired* t* test, **P < 0.01, ***P < 0.001, compared with the CHIR99021(-) group

To further prove that JAML promotes tumor progression by activating the Wnt/β-catenin pathway in LUAD cells, CHIR99021, an activator specific for Wnt/β-catenin, was uesd to perform rescue experiments in A549 cells. Our results showed that CHIR99021 could rescue expression changes of β-catenin, cyclin D1, survivin, E-cadherin, N-cadherin, vimentin, MMP2 and MMP9 caused by JAML knockdown in A549 cells (Fig. 8a). EdU and transwell assays confirmed that CHIR99021 can partially rescue the repression of proliferation, migration and invasion caused by silencing JAML in A549 cells (Fig. 8b–e). Together, these results suggest that JAML exerts most of functions via the Wnt/β-catenin pathway.

## Discussion

The highlight of our study is demonstration of the tumorigenic role of JAML in LUAD both in vivo and in vitro, as well as elucidation of the potential mechanisms in regulating the progression of LUAD. Our studies show that the expression of JAML is elevated in LUAD and that overexpression of JAML is positively related to pT and pTNM (Fig. 1, Table 6). We discovered that JAML plays an oncogenic role in LUAD. Reduction of JAML expression was associated with repression the abilities of proliferation, migration and invasion in LUAD cells. Correspondingly, overexpression of JAML was associated with increasion in proliferation, migration, and invasion in LUAD cells (Figs. 2, 3). JAML knockdown also mediated cell cycle arrest in G_0_/G_1_ phase and promoted cell apoptosis (Fig. 4). To in-depth clarify the actual function of JAML in tumorigenesis in vivo, a subcutaneous xenograft tumor model in nude mice with LUAD cells was established. The results show that overexpression of JAML significantly increased the xenograft tumor volume and vice versa (Fig. 5). Thus, we aimed to explore the possible potential mechanisms to explain how JAML plays an oncogenic role in LUAD. First, we discovered the effects of JAML on the MMPs, apoptotic-related proteins and hallmarks of EMT, the results demonstrated that silencing of JAML concomitant with a reduction in MMP2, MMP9, Bcl2, N-cadherin and vimentin, and an increase in Bax and E-cadherin and vice versa (Fig. 6). The Wnt/β-catenin pathway is a major pathway that can participate in complex malignant biological behaviors in various tumors. Next, the changes in key proteins of the pathway were analyzed in detail. It was found that the silencing of JAML was associated with a decrease in β-catenin, survivin and cyclin D1 and vice versa (Fig. 7). Rescue experiments showed that CHIR99021 could rescue the inhibition of EMT and MMP2, MMP9 expression caused by JAML knockdown in A549 cells (Fig. 8). EdU and transwell assays also confirmed that CHIR99021 could rescue the repression of proliferation, migration and invasion caused by JAML knockdown in A549 cells (Fig. 8). These results suggest that JAML exerts most of functions via the Wnt/β-catenin pathway. JAML may be a predictive biomarker and novel therapeutic target in LUAD.

Previous studies have shown that JAML promotes tumor progression in gastric cancer in vitro [18]. Since LUAD and most gastric cancers originate from epithelial cells, they may have a similar pathogenesis. Recent studies noted that the expression of JAML was decresed in LUAD [19, 20], which was different from our results. However, there were no sufficient experimental supports to confirm their conclusions. The main reason for this difference might be that most of their conclusions were obtained from online databases. As we know that tumor purity of surgical tumor specimens are often impure, the nontumor cells, such as immune cells and stromal cells dilute the tumor purity and influence on the genomic analysis of tumour samples [21, 22]. In addition, such findings may be also attributed to racial/ethnic differences.

Anti-apoptotic properties are common features of most cancers. Bcl2 family members consist of antiapoptotic proteins, such as Bcl2 and BclXL, and proapoptotic proteins, such as Bax and Bad [23]. Survivin can suppress apoptosis and promote cell division in various cancers [24, 25]. Our studies showed that overexpression of JAML tends to inhibit apoptosis through upregulation of Bcl2 and survivin and downregulation of Bax.

During the tumor progression and metastasis stages, EMT is a key process by which epithelial cells become detached since they lose their polarity due to the reduction of their expression of E-cadherin and increase in mesenchymal characteristics due to increased expression of N-cadherin and vimentin [26–30]. Thus, elucidating the underlying EMT mechanism is critical for tumor progression research. The Wnt/β-catenin pathway accounts for a large proportion of EMT among many signaling pathways [31]. β-catenin plays a central role in tumorigenesis in the Wnt/β-catenin signaling pathway [32, 33]. Our studies found that JAML is associated with EMT. Both EMT phenotype and MMP2, MMP9 expression could be rescued by an activator specific for Wnt/β-catenin. JAML exerts most of functions by activiting the Wnt/β-catenin signaling pathway.

The shortcomings of our study should be acknowledged. First, we did not have prognostic information for LUAD patients. As a result, the prognostic value of JAML in LUAD is still unknown. Second, although we propose that JAML may affect the Wnt/β-catenin pathway, multiple mechanisms are likely to govern JAML-mediated oncogenic functions and may act on any given gene, and the exact detailed mechanism by which JAML regulates the Wnt/β-catenin pathway was not identified in our study. Third, JAML is considered to be associated with immune infiltration. However, the experiments in this study did not involve immune-related aspects. Thus, further research on JAML is also needed.

## Conclusion

JAML is an oncogene in LUAD. Based on the in vivo and in vitro levels, JAML deficiency attenuated malignant biological behaviors in LUAD cells, including proliferation, migration and invasion. Overexpression of JAML enhanced the abovementioned malignant biological behaviors in LUAD cells, and JAML knockdown also mediated cell cycle arrest and promoted cell apoptosis. These effects may be associated with the Wnt/β-catenin pathway. Therefore, JAML may be a promising predictive biomarker in LUAD. Future strategies for clinical applications should antagonize the function of JAML to offer a novel target for LUAD.

## Supplementary Information

**Additional file 1: Figure S1** Expression of JAML in LUAD cells. **a** The expression of JAML in LUAD cell lines (A549, H1299, PC9, H1975). The internal reference gene was β-actin. **b** Quantitative analysis of (**a**) n = 3, unpaired *t* test. **c** Immunofluorescence assay showed JAML expression on cytoplasm of LUAD cells. **d–e** Knockdown efficiency of JAML in A549 (**d**) and H1299 cells (**e**) were confirmed after transient transfection of SiRNA for 72 h by Western blotting. The internal reference gene was β-actin. Quantitative analysis of n = 3, unpaired *t* test, **P < 0.01, ***P < 0.001, compared with siNC group. **f–g** Quantitative analysis of JAML overexpression in PC9 (**f**) and H1975 cells (**g**) after transfection with plasmid for 72 h. n = 3, unpaired *t* test, ***P < 0.001 compared with vector group.

## Data Availability

The datasets used and/or analyzed during the current study are available from the corresponding author on reasonable request.

## Abbreviations

LUAD: Lung adenocarcinoma
JAML: Junctional adhesion molecule-like protein
qRT-PCR: Quantitative real-time polymerase chain reactions
siRNA: Small interfering RNA
EdU: 5-Ethynyl-2′-deoxyuridine
CCK-8: Cell counting kit-8
JAMs: Junctional adhesion molecules
Ig: Immunoglobulin
JAM-A: Junctional adhesion molecule-A
JAM-B: Junctional adhesion molecule-B
JAM-C: Junctional adhesion molecule-C
JAM-4: Junctional adhesion molecule-4
DMEM: Dulbecco’s modified eagle’s medium
FBS: Fetal bovine serum
RIPA: Radioimmunoprecipitation assay
SDS–PAGE: Sodium dodecyl sulfate–polyacrylamide gel electrophoresis
PVDF: Polyvinylidene difluoride
TBST: Tris-buffered saline-tween
RT: Room temperature
ECL: Enhanced chemiluminescence
IHC: Immunohistochemistry RNAi: RNA interference
CDS: Coding sequence
EDTA: Ethylenediaminetetraacetic acid
DAB: 3,3 N-Diaminobenzidine tertrahydrochloride
IPP: Image pro plus
OE: Overexpression
NC: Negative control
BSA: Bovine serum albumin
PBS: Phosphate-buffered saline
DAPI: 4′,6-Diamidino-2-phenylindole
DNA: Deoxyribonucleic acid
MOI: Multiplicity of infection
PI: Propidium iodide
FITC: Fluorescein isothiocyanate
ARRIVE: Animal Research: Reporting of In Vivo Experiments
SEM: Standard error of the mean
TNM: Tumor-node-metastasis, system for staging cancer
MMP: Matrix metalloproteinase
EMT: Epithelial-to-mesenchymal transition
