# Supplementary figures and images for: Junctional adhesion molecule-like protein promotes tumor progression via the Wnt/β-catenin signaling pathway in lung adenocarcinoma
Source: J Transl Med. 2022 Jun 7;20:260. doi: 10.1186/s12967-022-03457-w (PMC9171988; doi:10.1186/s12967-022-03457-w)

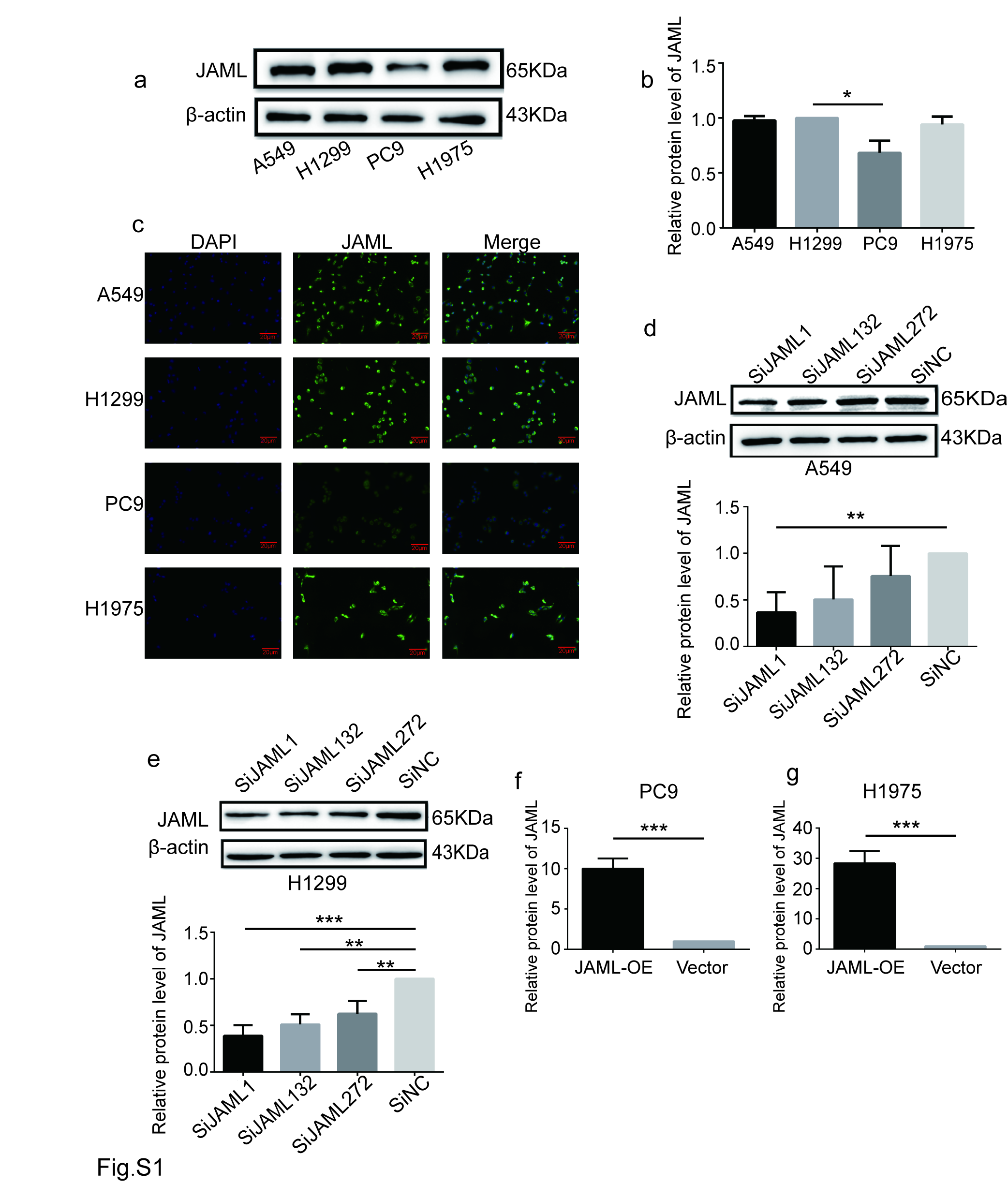

Supplement: Supplementary file 1 — Additional file 1: Figure S1 Expression of JAML in LUAD cells. a The expression of JAML in LUAD cell lines (A549, H1299, PC9, H1975). The internal reference gene was β-actin. b Quantitative analysis of (a) n = 3, unpaired t test. c Immunofluorescence assay showed JAML expression on cytoplasm of LUAD cells. d–e Knockdown efficiency of JAML in A549 (d) and H1299 cells (e) were confirmed after transient transfection of SiRNA for 72 h by Western blotting. The internal reference gene was β-actin. Quantitative analysis of n = 3, unpaired t test, **P < 0.01, ***P < 0.001, compared with siNC group. f–g Quantitative analysis of JAML overexpression in PC9 (f) and H1975 cells (g) after transfection with plasmid for 72 h. n = 3, unpaired t test, ***P < 0.001 compared with vector group. [file 12967_2022_3457_MOESM1_ESM.tif]
